# Reading ability underlies the composite effect for Arabic words

**DOI:** 10.1177/03010066251364208

**Published:** 2025-08-14

**Authors:** Rayan Kouzy, Zahra Hussain

**Affiliations:** American University of Beirut, Lebanon; École Normal Supérieure - PSL University, France; American University of Beirut, Lebanon; University of Plymouth, UK

**Keywords:** expertise, composite, orthography, Gestalt, connectedness, reading

## Abstract

The composite effect, originally demonstrated for faces, has recently been shown to suggest holistic processing of words. The effect is associated with reading fluency in Latin script, but not in nonalphabetic Chinese script, suggesting that script properties influence its relationship with reading expertise. We measured the composite effect for Arabic, a visually complex alphabetic script that offers a useful contrast against Latin and Chinese. Arabic-English bilinguals ( N= 24), and English-only readers ( N= 22) completed a composite effect task, in which they judged whether the left or right halves of word pairs were the same or different. The unattended half was either congruent or incongruent with the judgement, and the halves were presented in aligned or misaligned blocks. The composite effect, a reduction in the effect of congruency when the halves are misaligned, typically is interpreted as evidence for holistic processing. Arabic-English readers showed the composite effect for Arabic words, whereas English-only readers did not. Both groups showed the effect for English words. The effect size for the two scripts was equivalent in Arabic-English readers. These findings suggest that the composite effect for Arabic words, like that of Latin script words, requires the ability to read the script. Graphemic complexity or the cursive property of the script appears not to play a role in the composite effect in skilled readers.

## Introduction

The composite effect—first shown for faces, and then for other expertly recognized objects—is evidence for the processing of object features as interconnected wholes rather than as separate parts, also termed holistic processing (Boggan et al., 2012; Bukach et al., 2010; Cheung et al., 2008; Diamond & Carey, 1986; Farah et al., 1998; Feizabadi et al., 2021; Gauthier & Tarr, 2002; Jacques & Rossion, 2009; Kuefner et al., 2010; Richler et al., 2012). For example, recognizing the top half of a familiar face is difficult when it is paired with an unfamiliar bottom half, suggesting the two halves are not processed independently (Cheung et al., 2008). Printed words also elicit the effect, where words are split into their left and right halves, and readers struggle to recognize that the left halves of two words are identical if the right halves differ (Ventura et al., 2017, 2020b; Wong et al., 2012, 2011, see Anstis (2005) for a variation in which words were split top/bottom). This effect, termed the word composite or composite word effect, is thought to signify holistic word recognition, albeit at a later or more abstract stage of processing than that shown for faces (Ventura et al., 2017, 2020b). For Latin script, the word composite effect is consistent with phenomena such as the word superiority effect and letter transposition effects, which suggest whole-word recognition in fluent readers (Cattell, 1886; Reicher, 1969; Schoonbaert & Grainger, 2004; Wheeler, 1970). The goal of the work reported here was to examine the role of reading ability in holistic processing of Arabic words. Like Latin, Arabic is an alphabetic script, but it differs in other properties including visual complexity, word morphology, and omission of vowel sounds in writing. As we discuss below, such differences make it a useful comparison point for understanding which script properties underpin the association between reading fluency and the recognition processes revealed by composite effects.

With few exceptions (Brady et al., 2021), composite effects for Latin-script words are linked to expertise or reading fluency (Ventura et al., 2020b; Wong et al., 2011). For instance, the effect is stronger in native English readers than in second-language English readers (Wong et al., 2011), and for Portuguese words, it correlates with an independent measure of reading fluency, the word frequency effect (Ventura et al., 2020b). In contrast, composite effects for Chinese characters tend to be stronger in novices than in experts (Hsiao & Cottrell, 2009, but see Wong et al., 2012), and more pronounced in dyslexic than in typical readers (Tso et al., 2021, 2020). This suggests that the holistic process shown by this measure is not a universal aspect of reading expertise. Logographic scripts like Chinese may involve distinct perceptual and cognitive processes compared to alphabetic scripts (e.g., Tan et al., 2005; Tso, 2014). However, Chinese script is visually more complex than Latin (e.g., more within-character convolutions, Pelli et al., 2006), which may introduce differences in recognition strategies between scripts. Arabic is useful in this context because, like Latin, it is an alphabetic script, but it is as visually complex and inefficient to read as Chinese (Pelli et al., 2006). At minimum, Arabic provides a test of whether reading expertise elicits the composite effect across alphabetic script types.

The cursive nature of Arabic script offers additional insight into composite effects more broadly. Arabic letters typically are connected within words and their shapes vary by position. Hence, Arabic script is more configurational, and potentially Gestalt-like, than Latin script, where letter shape remains constant in printed text. Some evidence suggests that Gestalt properties alone can produce composite effects: untrained observers showed the composite effect for abstract line patterns with features like connectedness and closure, suggesting that holistic processing depends more on object properties than on observer expertise (Zhao et al., 2016). If so, non-Arabic readers may show composite effects for Arabic words.

In this experiment, we measured the composite effect for Arabic and English words in two groups: bilingual Arabic-English readers, and English-only readers. If reading ability drives the composite effect regardless of script complexity, it should appear for Arabic words only in bilinguals and for English words in both groups. This would suggest that visual complexity plays little role in the differences observed between Latin and Chinese scripts, and that expertise drives the effect generally for alphabetic scripts. If configurational features are the main factor, the composite effect should emerge in both groups—regardless of reading ability—and be larger for Arabic words than English words.

**Figure 1.**
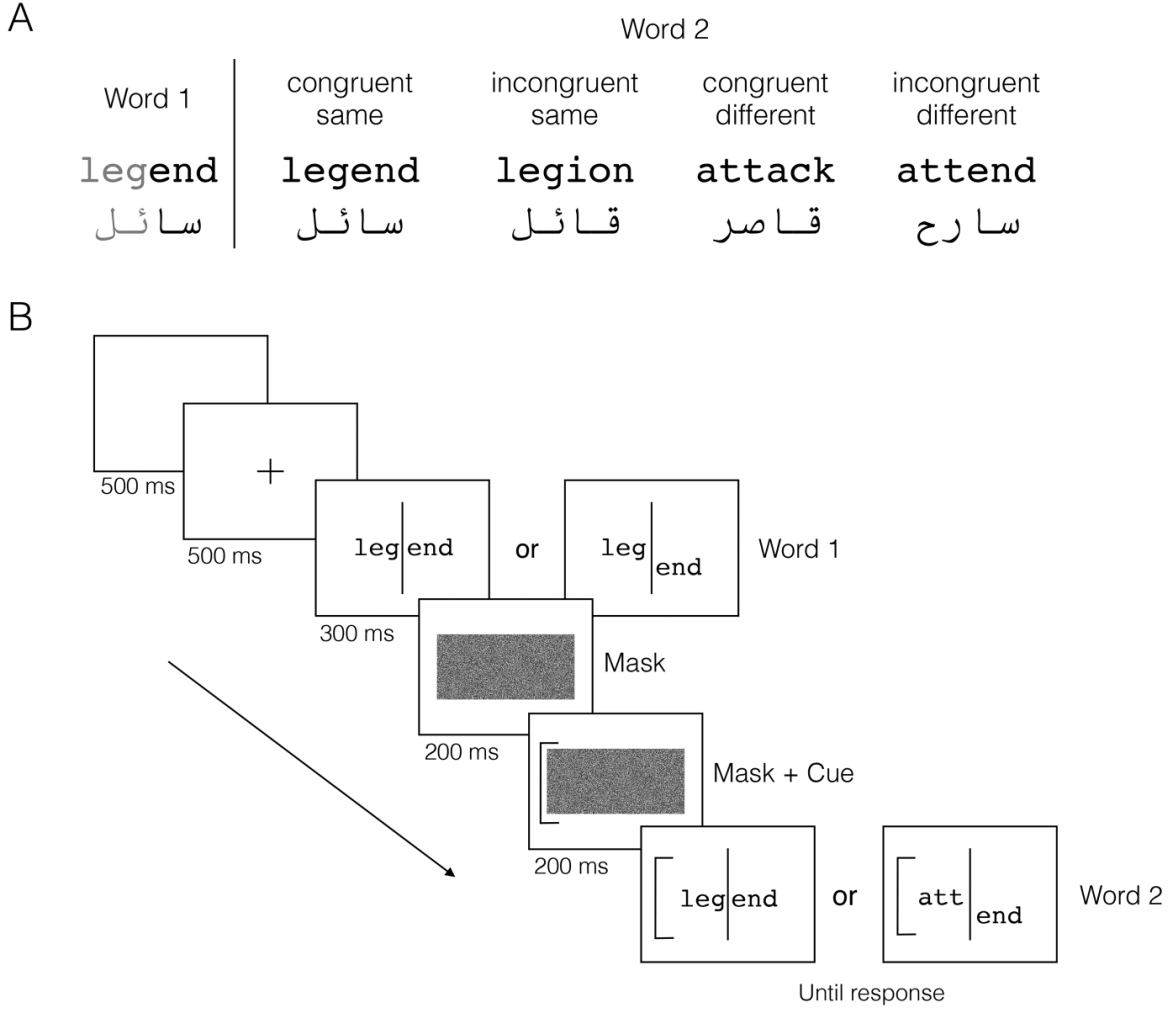
(A) Examples of the English and Arabic word stimuli used in the word composite task. The target half (left) is shown in light gray, not shaded in the experiment. (B) Schematic diagram of the task in the aligned (congruent-same; left) and misaligned (incongruent-different; right) conditions.

To measure the composite effect, we used the complete design, in which the effect is defined as an interaction between congruency and alignment in two successively presented objects (Richler & Gauthier, 2014) or words (Wong et al., 2011). Congruency refers to whether the two halves of the words yield the same response (e.g., legend-legend or legend-attack, see Figure 1; note that word halves comprise the left and right halves, rather than top and bottom. We discuss the implications of this method later). Alignment refers to whether the halves are visually offset, reducing the impact of congruency. Hence, the composite effect is the reduction in the congruency effect when the words are misaligned, or the decrease in whole-word interference in part recognition—a defining aspect of holistic processing. Our interest was in the four-way interaction of group, script-type, congruency and alignment, which would reveal whether the composite effect is specific to scripts that observers can read.

## Methods

### Design

The experimental design was a 2 (congruency: congruent vs. incongruent, within subjects) × 2 (alignment: aligned vs. misaligned, within subjects, blocked) × 2 (script: Arabic vs. English, within subjects, blocked) × 2 (group: English-only readers vs. Arabic-English bilingual readers, between subjects) mixed factorial design.

### Ethics Statement

All procedures of the experiment were approved by the Institutional Review Board for the Protection of Human Participants in Research and Research Related Activities (IRB) at the American University of Beirut, and the Ethics Board at McMaster University, Canada. All experiments were performed in accordance with relevant guidelines and regulations. Informed consent was obtained from all subjects before they participated in the study.

### Subjects

The final sample comprised 22 monolingual English readers (mean age = 19.4 years; six males) and 24 bilingual Arabic-English readers (mean age = 19.6 years; 13 males), drawn from an initial pool of 26 monolingual and 30 bilingual subjects. This sample size is similar to previous studies examining between-group variations in the composite effect (e.g., Hsiao & Cottrell, 2009; Wong et al., 2011). Monolingual subjects were native English-speaking undergraduate students at McMaster University, Canada, with no experience in reading, writing, or speaking Arabic. Bilingual subjects were undergraduate students at the American University of Beirut, whose native language was Arabic, and who had received formal education in both English and Arabic. Bilingual subjects completed a self-report proficiency measure (scale: 0–4) assessing reading, writing, and speaking abilities in both languages. To be included, they needed an average score above 2 in each language (mean Arabic rating = 4.4, *SD* = 0.8; English = 4.1, *SD* = 0.8; mean difference between languages = 0.3, *SD* = 1). Four bilingual subjects were excluded due to low Arabic proficiency, and data from one subject were missing or not saved correctly. Additionally, four monolinguals and one bilingual subject were excluded from analyses for performing at chance level (below 60% correct) across stimulus conditions in one or both languages. All subjects had normal or corrected-to-normal vision, assessed using the Early Treatment of Diabetic Retinopathy Study (ETDRS) acuity chart. Subjects received either course credit or monetary compensation for their time.

### Task

In the word composite task, participants judge whether the first or last few letters of two successively presented words are the same or different (Figure 1). For the English words in Figure 1, the target halves—the left halves or first three letters—are indicated by a cue (a square bracket) next to the second word. Participants must determine whether the cued halves match across the two presentations. Congruent pairs are those where both halves either match or differ, yielding a common response (same or different). Thus, congruent-same pairs are identical words (e.g., legend-legend), and congruent-different pairs have no letters in common at each letter position (e.g., legend-attack). Incongruent pairs contain conflicting information between the target and irrelevant halves: incongruent-same pairs have identical target halves but different irrelevant halves (e.g., legend-legion). Incongruent-different pairs have differing target halves but identical irrelevant halves (e.g., legend-attend). The congruency effect is the difference in accuracy or response time between congruent and incongruent conditions, reflecting interference from the irrelevant halves. A composite effect exists if the congruency effect is reduced when the word halves are misaligned (offset from each other). In a complete design, the composite effect is a 2 (congruency) × 2 (alignment) interaction effect (Richler & Gauthier, 2014). In partial designs, congruency effects are not measured (only same-incongruent and different-congruent trials are used, with the uncued half always different), and the composite effect corresponds to the main effect of alignment (e.g., Kuefner et al., 2010). In another variation, the congruency effect alone serves as an indicator of holistic or configurational processing (Brady et al., 2021; Lewis & Hills, 2018; Wong et al., 2011).

### Stimuli

For each script, six sets of four words (common nouns and verbs) were used to generate word pairs for the congruent-same, incongruent-same, congruent-different, and incongruent-different conditions (see Supplemental Table 1). Within each set, these conditions were created by manipulating the correspondence between the left and right halves of the words, as described above. English words were six letters long and Arabic words were four letters long. Shorter words were used for Arabic because the stimulus conditions were more difficult to generate with longer words. Each script contained 24 words, all of which met the four conditions when either the first or second half was cued. Congruent-same and congruent-different pairs remained unchanged regardless of which half was cued. For example, legend-legend (congruent-same) and legend-attack (incongruent-different) were valid for both cued halves. Incongruent-same and incongruent-different pairs were swapped depending on the cued half. For example, legend-legion (incongruent-same) and legend-attend (incongruent-different) were used when the left half was cued, and legend-attend (incongruent-same) and legend-legion (incongruent-different) were used when the right half were cued.

Stimuli were presented using PsychoPy3, Version 3.1.5 (Peirce, 2008) on monitors with a 60 Hz frame rate and a resolution of 1024 × 768 pixels. At McMaster University, stimuli were generated on an iMac and displayed on an NEC MultiSync FE992 monitor. At the American University of Beirut, stimuli were generated on an Intel Skull Canyon NUC computer and displayed on Dell monitors (Dell M783S and M783P). English words were displayed in Courier font and subtended 2 ∘ × 6.6 ∘ from a viewing distance of 114 cm. Arabic words (presented without diacritics) were also in Courier font, subtending 2 ∘ × ≈ 4.25 ∘ of visual angle. In the aligned condition, all letters were presented at the same *y*-coordinate. In the misaligned condition, the second half of each word was lowered by 1 ∘ of visual angle (right half for English, left half for Arabic; see Figure 1). A vertical line (1.5 pixels wide, 5 ∘ long) was drawn between the two halves in both conditions. Arabic words were misaligned without altering letter shape at the breakpoint. To achieve this, words were printed twice on the screen (one below the other), and masked with the background color on either half, revealing a single word with misaligned halves. Masks were placed at the midpoint between the second and third letter to preserve letter shape.

### Procedure

Subjects were seated at an adjustable chin rest 114 cm from the monitor. Each trial began with a blank screen (500 ms), followed by a fixation point (500 ms), and a centrally presented word (300 ms). The word was then replaced by a mask (200 ms), after which a cue (a square bracket) appeared on either the left or right side of the fixation point for 200 ms. Immediately after the mask disappeared, a second word appeared alongside the cue, and both remained on the screen until the participant had responded. Participants were instructed to report whether the cued halves of the words were the same or different, responding via keypress. Auditory feedback was provided: a high-pitched tone for correct responses and a low-pitched tone for incorrect responses. Each session lasted approximately 40 min and included trials with both Arabic and English words. The session was divided into four randomized blocks: English aligned, English misaligned, Arabic aligned, and Arabic misaligned. The congruency conditions were randomized within the block, and word pair order was randomized within each trial. Monolingual participants tested in Canada completed 288 trials per block (6 sets × 2 congruency conditions × 2 trial type (same vs. different) × 2 cued sides × 6 repetitions per condition). Bilingual participants, tested in Lebanon, completed 240 trials (5 repetitions per condition), due to constraints on experiment duration.

The first 10 trials for every subject were treated as practice trials and were excluded from the analyses. Additionally, trials with response times shorter than 100 ms or longer than 3 s were removed (accounting for 2% of all trials). Two dependent measures were analyzed: sensitivity ( $d^{\prime }$ ) and response time on correct trials. $d^{\prime }$ was calculated by treating same-pair trials as signal trials (hits were responses of “same” in same trials, false alarms were responses of “same” in different trials). Calculations were performed separately for each word set to allow word to be treated as a random factor in subsequent analyses. $d^{\prime }$ was computed for each of the eight stimulus conditions (2 congruency × 2 alignment × 2 language), averaged over cue side (i.e., ≈ 12 trials per proportion correct estimation after practice and excluded trials were removed). $d^{\prime }$ was calculated as below:

*d*′ = *z*(*p*(“same” | same))- z(p(“same” | different)) (1)

Additionally, $d^{\prime }$ was calculated using the independent observation model for same/different tasks (Macmillan et al., 2005). In rare cases, where $pc_{max}$ was below 0.5, this formulation yielded incomputable values, but the overall pattern remained consistent. Therefore, we report $d^{\prime }$ using the standard formula.

## Results

$d^{\prime }$ and response times were analyzed using R (Version 4.1.2, R Core Team, 2020) and R Studio (Version 1.4.17) using linear mixed-effects models from the lme4 and lmerTest packages (Bates et al., 2015; Kuznetsova et al., 2017). These models are well-suited for designs that incorporate both fixed effects (experimental conditions) and random effects (e.g., subjects and items). The models included congruency, alignment, and language as within-subjects fixed effects, group as a between-subjects fixed effect, and subject and word set as random effects. All interactions between the fixed effects were included, while interactions involving subject were included up to the highest-order interaction, mirroring a repeated-measures ANOVA approach. Random effects with variance components of zero were excluded from the final model.

As noted earlier, the composite effect is shown by a significant 2 × 2 (congruency × alignment) interaction. Higher-order interactions involving language, group, or both with congruency and alignment indicated whether the composite effect varied across these factors. Our focus was on the four-way interaction (group × language × congruency × alignment), which would confirm whether the composite effect was obtained selectively for a specific group in a given language. If this four-way interaction was significant, the three-way interaction (congruency × alignment × group) was analyzed separately for each language. If the three-way interaction was significant within a language, the congruency × alignment interaction was then examined separately for each group within that language, with Bonferroni corrections applied to control for family-wise Type 1 error.

Response time analyses were conducted on the raw data from correct trials only (38,756 trials across 46 subjects). $d^{\prime }$ analyses were done on aggregated data as described above. Additionally, parallel analyses were conducted on two supplementary dependent measures—accuracy, and the nonparametric sensitivity measure $A^{\prime }$ —both of which yielded results consistent with those obtained for $d^{\prime }$ and response time. Hence, those additional analyses are not reported.

### Sensitivity ( $d^{\prime }$ )

**Figure 2.**
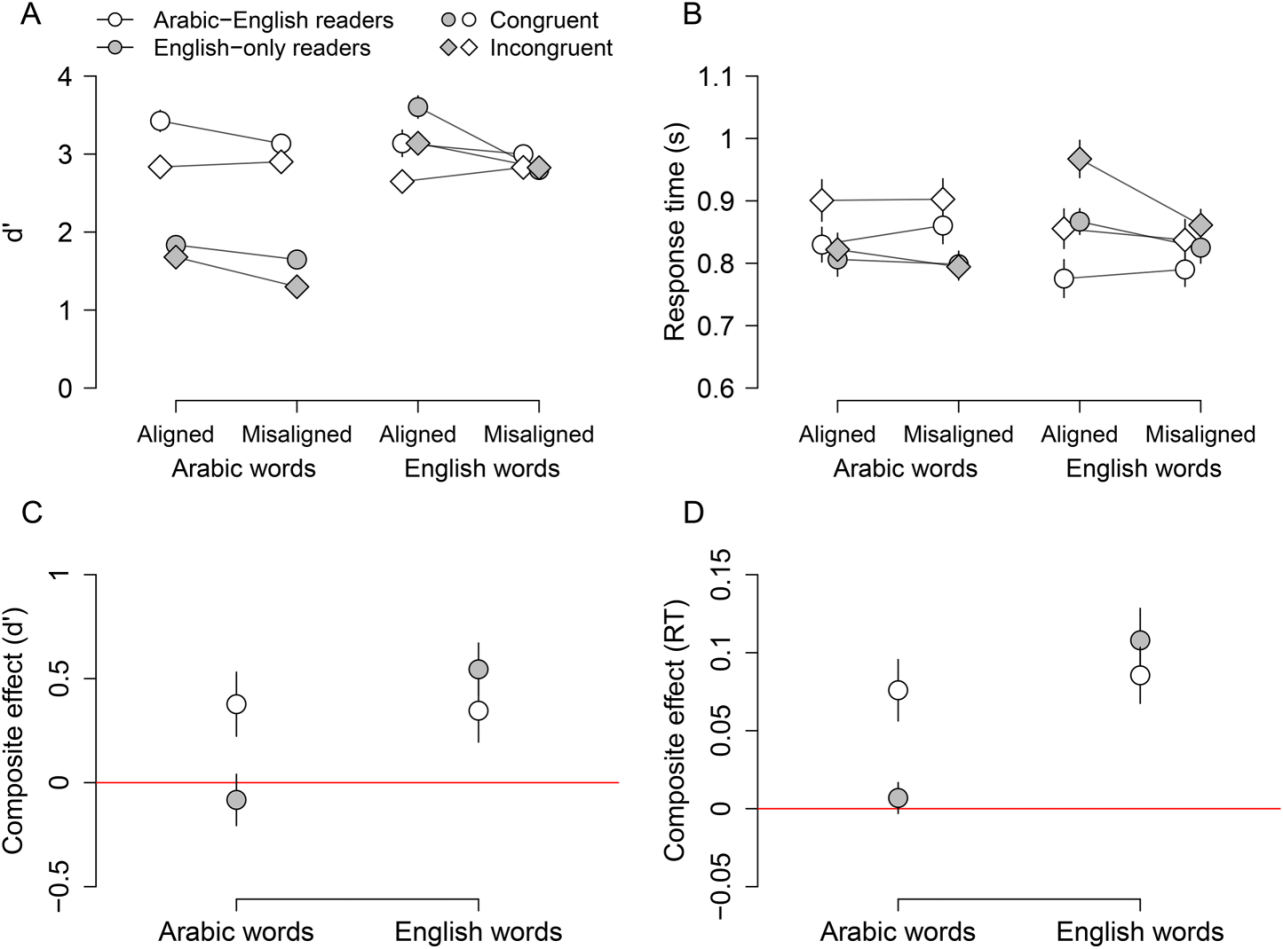
Performance on the word composite task. (A, B) $d^{\prime }$ and response times on correct trials in the eight stimulus conditions for the two groups. Open symbols represent the Arabic-English readers; filled symbols show the English-only readers. Circles and diamonds correspond to the congruent and incongruent conditions, respectively. Error bars show within-subjects standard error of the mean (Cousineau, 2005). (C, D) The composite effect computed as the difference between the congruency effect (congruent–incongruent) in the aligned and misaligned conditions, for each language for each group.

Figure 2A shows $d^{\prime }$ for the two groups across the eight stimulus conditions (2 congruency × 2 alignment × 2 language). A composite effect is evident for Arabic words in Arabic-English readers, as indicated by a larger congruency effect in the aligned condition than in the misaligned condition. This effect does not appear for English-only readers with Arabic words. However, for English words, a composite effect is present for both groups. Furthermore, Arabic-English readers showed higher $d^{\prime }$ for Arabic words compared to English-only readers. Figure 2C shows the average composite effect for each language for each group. The composite effect was calculated for each subject as the difference between the congruency effects in the aligned and misaligned conditions. This plot confirms that the composite effect was present for Arabic words only in Arabic readers but for English words in both groups. Furthermore, the size of the composite effect appears similar for Arabic and English words in Arabic-English readers.

The omnibus mixed-effects model revealed significant main effects of group, $F(1,44)=7.89,p=.007,\eta_{\;p}^{2}=0.15$ , language, $F(1,32.67)=37.79,p<.001,\eta_{\;p}^{2}=0.54$ , congruency, $F(1,44)=13.78,p<.001,\eta_{\;p}^{2}=0.24$ , and alignment, F(1,96.58)=10.83, $p=.001,\eta_{\;p}^{2}=0.10$ . There was a significant four-way interaction between group, language, congruency and alignment, $F(1,1874.85)=7.51,p=0.006,\eta_{\;p}^{2}=.004$ , which indicated that the composite effect (congruency × alignment) depended on language and group. Several lower-order interaction effects also were significant (group × language, group × alignment, congruency × alignment, language × congruency × alignment), but only the highest-order interaction was further analyzed. Follow-up models evaluated the effects of congruency, alignment and group separately for each language using the same fixed and random effects as in the omnibus model.

**Arabic words:** The three-way interaction between group, congruency and alignment was significant, $F(1,959)=8.27,p=.004,\eta_{\;p}^{2}=0.008$ , as well as the main effects of group, $F(1,44)=43.83,p<.001,\eta_{\;p}^{2}=0.50$ , congruency, $F(1,44)=13.99,p<.001,\eta_{\;p}^{2}=0.24$ , and alignment, $F(1,44)=6.38,p=.012,\eta_{\;p}^{2}=0.13$ . The three-way interaction indicated that the composite effect for Arabic words depended on group. Further analyses showed that for Arabic readers, there was a significant congruency × alignment interaction, $F(1,498)=6.22,p=.012,\eta_{\;p}^{2}=0.01$ , confirming a composite effect consistent with the pattern observed in Figure 2A (there also was a significant main effect of congruency, $F(1,23)=7.008,p=.014,\eta_{\;p}^{2}=0.23$ , for this group). For English-only readers, the congruency × alignment interaction was not significant, F(1,456)=2.40,p=.12 , but there were significant main effects of congruency, $F(1,21)=11.47,p=.0027,\eta_{\;p}^{2}=0.35$ , and alignment, $F(1,21)=9.71,p=.005,\eta_{\;p}^{2}=0.32$ . Thus, the Arabic-English readers showed a composite effect for Arabic words, whereas the English readers did not.

**English words:** The three-way group × congruency × alignment interaction was not significant, F(1,959)=0.87,p=.35 , but a significant two-way congruency × alignment interaction was found, $F(1,959)=19.31,p<.001,\eta_{\;p}^{2}=0.02$ , confirming a composite effect for English words for both groups, consistent with Figure 2A. Other significant effects included the main effect of congruency, $F(1,44)=9.18,p=.0044,\eta_{\;p}^{2}=0.17$ , and alignment, $F(1,44)=5.93,p=.01,\eta_{\;p}^{2}=0.12$ , and the group × alignment interaction, $F(1,44)=6.86,p=0.012,\eta_{\;p}^{2}=0.14$ . Follow-up analyses revealed that the group × alignment interaction resulted from English-only readers performing significantly better in the aligned than misaligned condition, F(1,21)=12.03,p=.002 , whereas Arabic-English readers showed no significant difference between alignment conditions, F(1,23)=0.02,p=.89 .

**Arabic versus English words (bilingual readers only)** A separate model tested whether the composite effect differed between Arabic and English words in Arabic-English readers. Such a difference would be reflected in a congruency × alignment × language interaction. There was a significant main effect of congruency, $F(1,23)=6.39,p=.018,\eta_{\;p}^{2}=0.22$ , and a significant congruency × alignment interaction, $F(1,23)=6.36,p=.019,\eta_{\;p}^{2}=0.22$ . The congruency × alignment × language interaction was not significant, F(1,23)=0.03,p=.85 , indicating that the size of the composite effect did not differ between Arabic and English words in Arabic-English readers.

In summary, a composite effect was observed for Arabic words in bilingual readers but not in English-only readers. For English words, a composite effect was found in both groups. The size of the composite effect did not differ between Arabic and English words for the bilingual readers.

### Response Time

Figure 2B presents mean response times across the eight stimulus conditions for the two groups. The congruency effect is evident, with faster response times in congruent than incongruent conditions. Similar to $d^{\prime }$ , Figure 2B suggests that a composite effect were present for Arabic words in Arabic-English readers only, whereas for English words, a composite effect was present for both groups. Figure 2D illustrates the average composite effect in response times for each condition, calculated for each subject as the difference between the congruency effect in the aligned and misaligned conditions. As with $d^{\prime }$ , the composite effect was positive in three out of four cases: for Arabic-English readers in both languages and for English readers with English words.

A linear mixed-effects model (identical in formulation to that used for $d^{\prime }$ ) revealed a significant main effect of congruency, $F(1,43)=44.95,p<.001,\eta_{\;p}^{2}=0.51$ , and a significant four-way interaction between group × language × congruency × alignment, F(1,38510)=3.89, $p<.05,\eta_{\;p}^{2}=0.0001$ . Additionally, the following interactions were significant: group × language, language × congruency, congruency × alignment, and group × language × congruency. As with $d^{\prime }$ , only the four-way interaction was decomposed. Separate models analyzed congruency, alignment and group effects within each language, using the same fixed and random effects as in the omnibus model.

**Arabic words:** The three-way interaction between congruency, alignment and group was not significant, F(1,18466.7)=1.01,p=.32 , but there was a significant group × congruency interaction, $F(1,43.6)=10.83,p=.002,\eta_{\;p}^{2}=0.20$ , a significant congruency × alignment interaction, $F(1,18467.3)=5.89,p=.01,\eta_{\;p}^{2}=0.0003$ , and a significant main effect of congruency, $F(1,43.6)=10.92,p=.002,\eta_{\;p}^{2}=0.20$ . The congruency × alignment interaction confirms the composite effect observed in Figure 2C. The group × congruency interaction indicates that the congruency effect was not significant for English-only readers, F(1,8965.8)=0.001,p=.99 , but it was significant for the Arabic-English readers, $F(1,9524.3)=57.37,p<.0001,\eta_{\;p}^{2}=0.006$ .

**English words:** There was a significant congruency × alignment interaction, $F(1,43.9)=20.45,p<.0001,\eta_{\;p}^{2}=0.001$ , and there was a significant main effect of congruency, $F(1,44.6)=63.37,p<.0001,\eta_{\;p}^{2}=0.59$ . No other main effects or interactions were significant ( F<4,p>.05 ). This pattern of results confirms that the composite effect was present for English words in both groups.

In summary, the response time results indicate a composite effect for Arabic words that was clearer for Arabic-English readers than English-only readers (for whom there was no significant effect of congruency). The composite effect for English words was observed in both groups. This pattern of results closely mirrors the findings for $d^{\prime }$ .

## Discussion

The word composite task is a same/different perceptual judgement of word halves that does not require reading ability. However, the word composite effect—a reduction in perceptual interference when word halves are misaligned—here depended on reading ability. Specifically, Arabic-English bilingual readers, but not English-only readers, showed a composite effect for Arabic words, despite both groups performing above chance on the task. Hence, the composite effect for words relies on familiarity with the letters, or the ability to read the script. To the extent that composite effects for words provide evidence for holistic word recognition, these results support holistic processing as a marker of word recognition skill for alphabetic scripts (Ventura et al., 2017, 2020b; Wong et al., 2012, 2011). Broader mechanisms associated with familiarity or expertise (e.g., attentional factors) also were evident, where English readers performed better overall with English words than with Arabic words. Such factors can only account for the results to the extent that they reduce the congruency effect specifically (rather than performance in both congruency conditions) under misalignment for that script (i.e., account for the interaction effect in addition to any main effects of language or alignment). Word or letter alignment-related accounts alone (e.g., reduced crowding under misalignment) also do not explain the results.

These results provide the first evidence of a composite effect for Arabic words, extending findings from other languages and scripts (Hsiao & Cottrell, 2009; Ventura et al., 2017; Wong et al., 2012, 2011). The results are consistent with the patterns observed in fluent readers of Latin-script languages (Ventura et al., 2020b; Wong et al., 2011), but contrast with findings from novice readers of Chinese characters (Hsiao & Cottrell, 2009; Tso, 2014; Tso et al., 2022, 2021). Given that Chinese and Arabic characters are similarly complex (as measured by perimetric complexity; Pelli et al., 2006), this difference in expertise effects may stem from the nature of the writing system itself. One explanation is that logographic scripts involve more analytical than holistic perceptual processing (Liu et al., 2022; Tan et al., 2001; Tien et al., 2023; Wang et al., 2019). This would imply that the mechanisms of reading expertise are less universal across variations in script than previously assumed (Rueckl et al., 2015). Alternatively, measures of letter complexity may overlook graphemic subtleties (e.g., variability in stroke orientation and position), which might better predict perceptual strategies across different scripts. Another possibility is that expertise-dependent composite effects in Chinese script may emerge under different stimulus conditions—for instance when compound characters rather than individual Chinese characters are used, or when words are split left-right rather than top-bottom (as by Wong et al., 2012, see further on this point below). Future work should examine the stimulus manipulations that produce expertise-dependent composite effects across writing systems.

Among Arabic-English bilingual readers—who on average were equally proficient in both scripts—the size of the composite effect did not differ between Arabic and English words. This suggests that given sufficient reading ability, the composite effect is not influenced by graphemic differences in alphabetic scripts. Furthermore, the connectedness or cursive nature of Arabic letters (a Gestalt-like property) did not elicit composite effects in nonreaders, in contrast to findings for abstract line patterns (Zhao et al., 2016). Hence, the effect appears not to be tied to the visual properties of the word form, and consistent with other studies may be rooted in higher-level lexical representations. Indeed, for Latin script, the effect is reduced or absent for pseudowords and is insensitive to variations in surface visual properties such as font or case (e.g., Ventura et al., 2020a, 2017; Wong et al., 2011). Additionally, the effect has been shown to depend on the phonological properties of the words, such that it is more robust for words with shallow orthography (consistent grapheme-phoneme mapping), than for words with ambiguous pronunciation (Ventura et al., 2019). These findings further suggest that the composite effect or the holistic process it suggests is influenced by linguistic properties beyond visual appearance. Follow-up experiments should probe the degree to which reading ability or lexical knowledge is required: for instance, is the effect obtained in readers simply familiar with the letters, or in sublexical readers who can sound out words without lexical or semantic knowledge?

The absence of letter form effects and the dominance of higher-order lexical effects in prior studies may be attributable to the specific manipulation used in the word composite task—splitting words left/right, rather than top/bottom as typically is done in face composite tasks. Left–right splitting preserves letter identity, potentially shifting the locus to higher-level representations and reducing word form-related effects. If words were split differently, composite effects might emerge for pseudowords and nonreaders as well. Incidentally, an early study found that readers failed to recognize when the upper halves of different words were identical while the lower halves differed (e.g., bag vs. hoe), suggesting that this type of holistic processing extends to word form under certain conditions (Anstis, 2005). However, that study did not test nonreaders or pseudowords, leaving open the question of whether the effect relied on reading ability. In general, it is conceivable that variations in the composite task or stimulus manipulations differentially invoke lexical knowledge or sensitivity to word form.

Word and face recognition typically are framed as distinct processes—one part-based and hierarchical (letter-by-letter recognition: McClelland, 1981; Rumelhart & McClelland, 1982), and the other holistic, sensitive to subtle configurational differences within a fixed arrangement of features (eyes over nose over mouth). Neural evidence supports this distinction, with words preferentially engaging left-lateralized regions of the ventral occipitotemporal cortex (Bouhali et al., 2014; Dehaene et al., 2005; Puce et al., 1996; Purcell et al., 2011; Wimmer et al., 2016), while faces are predominantly processed in the right fusiform gyrus and associated regions (Puce et al., 1996; Rossion et al., 2003; Yovel et al., 2008). From this perspective, composite effects for words—typically associated with holistic face perception—run against the grain.

A different view, however, acknowledges part-based and holistic mechanisms for both words and faces (Martelli et al., 2005; Pelli et al., 2003). By this account, feature recognition precedes holistic processing for faces and words alike, reconciling traditional models of reading and object recognition with composite-like effects (Barnhart & Goldinger, 2013). Familiarity or expertise enhances recognition and elicits effects associated with holistic processing, as seen here with Arabic words. Yet, familiarity does not eliminate the ability to recognize individual parts—readers clearly succeed at part-based judgements regardless of alignment–and the composite effect provides no evidence that it does.

## Supplemental Material

sj-pdf-1-pec-10.1177_03010066251364208 - Supplemental material for Reading ability underlies the composite effect for Arabic words Supplemental material, sj-pdf-1-pec-10.1177_03010066251364208 for Reading ability underlies the composite effect for Arabic words by Rayan Kouzy and Zahra Hussain in Perception

## References

[bibr1-03010066251364208] Anstis S. (2005). Last but not least. Perception, 34, 237–240. 15832573 10.1068/p5412

[bibr2-03010066251364208] Barnhart A. S. Goldinger S. D. (2013). Rotation reveals the importance of configural cues in handwritten word perception. Psychonomic Bulletin & Review, 20, 1319–26. 10.3758/s13423-013-0435-y 23589201 PMC3748233

[bibr3-03010066251364208] Bates D. Maechler M. Bolker B. M. Walker S. C. (2015). Fitting linear mixed-effects models using lme4. Journal of Statistical Software, 67, 1–48.

[bibr4-03010066251364208] Boggan A. L. Bartlett J. C. Krawczyk D. C. (2012). Chess masters show a hallmark of face processing with chess. Journal of Experimental Psychology. General, 141, 37–42. 10.1037/a0024236 21787101

[bibr5-03010066251364208] Bouhali F. Thiebaut de Schotten M. Pinel P. Poupon C. Mangin J. F. Dehaene S. Cohen L. (2014). Anatomical connections of the Visual Word Form Area. Journal of Neuroscience, 34, 15402–14. 10.1523/JNEUROSCI.4918-13.2014 25392507 PMC6608451

[bibr6-03010066251364208] Brady N. Darmody K. Newell F. N. Cooney S. M. (2021). Holistic processing of faces and words predicts reading accuracy and speed in dyslexic readers. PloS one, 16, e0259986. 10.1371/journal.pone.0259986 PMC8673614 34910756

[bibr7-03010066251364208] Bukach C. M. Phillips W. S. Gauthier I. (2010). Limits of generalization between categories and implications for theories of category specificity. Attention, Perception & Psychophysics, 72, 1865–74. 10.3758/APP.72.7.1865 PMC2957663 20952784

[bibr8-03010066251364208] Cattell J. (1886). The time it takes to see and name objects. Mind; A Quarterly Review of Psychology and Philosophy, 11, 63–65.

[bibr9-03010066251364208] Cheung O. S. Richler J. J. Palmeri T. J. Gauthier I. (2008). Revisiting the role of spatial frequencies in the holistic processing of faces. Journal of Experimental Psychology. Human Perception and Performance, 34, 1327–36. 10.1037/a0011752 19045978

[bibr10-03010066251364208] Cousineau D. (2005). Confidence intervals in within-subjects designs: A simpler solution to Loftus and Masson’s method. Tutorial in Quantitative Methods for Psychology, 1, 4–45.

[bibr11-03010066251364208] Dehaene S. Cohen L. Sigman M. Vinckier F. (2005). The neural code for written words: a proposal. Trends in Cognitive Sciences, 9, 335–41. 10.1016/j.tics.2005.05.004 15951224

[bibr12-03010066251364208] Diamond R. Carey S. (1986). Why faces are and are not special: An effect of expertise. Journal of Experimental Psychology. General, 115, 107–17. 10.1037//0096-3445.115.2.107 2940312

[bibr13-03010066251364208] Farah M. J. Wilson K. D. Drain M. Tanaka J. N. (1998). What is "special" about face perception? Psychological Review, 105, 482–98. 10.1037/0033-295x.105.3.482 9697428

[bibr14-03010066251364208] Feizabadi M. Albonico A. Starrfelt R. Barton J. J. S. (2021). Whole-object effects in visual word processing: Parallels with and differences from face recognition. Cognitive Neuropsychology, 38, 231–257. 10.1080/02643294.2021.1974369 34529548

[bibr15-03010066251364208] Gauthier I. Tarr M. J. (2002). Unraveling mechanisms for expert object recognition: Bridging brain activity and behavior. Journal of Experimental Psychology. Human Perception and Performance, 28, 431–46. 10.1037//0096-1523.28.2.431 11999864

[bibr16-03010066251364208] Hsiao J. H. Cottrell G. W. (2009). Not all visual expertise is holistic, but it may be leftist: The case of Chinese character recognition. Psychological Science, 20, 455–63. 10.1111/j.1467-9280.2009.02315.x 19399974

[bibr17-03010066251364208] Jacques C. Rossion B. (2009). The initial representation of individual faces in the right occipito-temporal cortex is holistic: Electrophysiological evidence from the composite face illusion. Journal of Vision, 9, 8.1–16. 10.1167/9.6.8 19761299

[bibr18-03010066251364208] Kuefner D. Jacques C. Prieto E. A. Rossion B. (2010). Electrophysiological correlates of the composite face illusion: Disentangling perceptual and decisional components of holistic face processing in the human brain. Brain and Cognition, 74, 225–38. 10.1016/j.bandc.2010.08.001 20851511

[bibr19-03010066251364208] Kuznetsova A. Brockhoff P. B. Christensen R. H. B. (2017). lmertest package: Tests in linear mixed effects models. Journal of Statistical Software, 82, 1–26.

[bibr20-03010066251364208] Lewis M. B. Hills P. J. (2018). Perceived race affects configural processing but not holistic processing in the composite-face task. Frontiers in Psychology, 9, 1456. 10.3389/fpsyg.2018.01456 30177898 PMC6109712

[bibr21-03010066251364208] Liu C. Y. Tao R. Qin L. Matthews S. Siok W. T. (2022). Functional connectivity during orthographic, phonological, and semantic processing of Chinese characters identifies distinct visuospatial and phonosemantic networks. Human Brain Mapping, 43, 5066–5080. 10.1002/hbm.26075 36097409 PMC9582368

[bibr22-03010066251364208] Macmillan N. A. Creelman C. D. (2005). Detection Theory: A User’s Guide. Second edition. Mahwah, NJ: Lawrence Erlbaum Associates Publishers.

[bibr23-03010066251364208] Martelli M. Majaj N. J. Pelli D. G. (2005). Are faces processed like words? A diagnostic test for recognition by parts. Journal of Vision, 5, 58–70. 10.1167/5.1.6 15831067

[bibr24-03010066251364208] McClelland J. L. R. D. (1981). An interactive activation model of context effects in letter perception: Part 1. An account of basic findings. Psychological Review, 88, 375–407. 7058229

[bibr25-03010066251364208] Peirce J. W. (2008). Generating stimuli for neuroscience using PsychoPy. Frontiers in Neuroinformatics, 2, 10. 10.3389/neuro.11.010.2008 19198666 PMC2636899

[bibr26-03010066251364208] Pelli D. G. Burns C. W. Farell B. Moore-Page D. C. (2006). Feature detection and letter identification. Vision Research, 46, 4646–74. 10.1016/j.visres.2006.04.023 16808957

[bibr27-03010066251364208] Pelli D. G. Farell B. Moore D. C. (2003). The remarkable inefficiency of word recognition. Nature, 423, 752–6. 10.1038/nature01516 12802334

[bibr28-03010066251364208] Puce A. Allison T. Asgari M. Gore J. C. McCarthy G. (1996). Differential sensitivity of human visual cortex to faces, letterstrings, and textures: A functional magnetic resonance imaging study. The Journal of Neuroscience, 16, 5205–15. 10.1523/JNEUROSCI.16-16-05205.1996 8756449 PMC6579313

[bibr29-03010066251364208] Purcell J. J. Napoliello E. M. Eden G. F. (2011). A combined fMRI study of typed spelling and reading. NeuroImage, 55, 750–62. 10.1016/j.neuroimage.2010.11.042 21109009 PMC3035733

[bibr30-03010066251364208] R Core Team . (2020) *R: A language and environment for statistical computing*. R Foundation for Statistical Computing, Vienna, Austria.

[bibr31-03010066251364208] Reicher G. (1969). Perceptual recognition as a function of the meaningfulness of stimulus material. Journal of Experimental Psychology, 81, 275–280. 5811803 10.1037/h0027768

[bibr32-03010066251364208] Richler J. J. Gauthier I. (2014). A meta-analysis and review of holistic face processing. Psychological Bulletin, 140, 1281–302. 10.1037/a0037004 24956123 PMC4152424

[bibr33-03010066251364208] Richler J. J. Palmeri T. J. Gauthier I. (2012). Meanings, mechanisms, and measures of holistic processing. Frontiers in Psychology, 3, 553. 10.3389/fpsyg.2012.00553 23248611 PMC3520179

[bibr34-03010066251364208] Rossion B. Caldara R. Seghier M. Schuller A. M. Lazeyras F. Mayer E. (2003). A network of occipito-temporal face-sensitive areas besides the right middle fusiform gyrus is necessary for normal face processing. Brain, 126, 2381–95. 10.1093/brain/awg241 12876150

[bibr35-03010066251364208] Rueckl J. G. Paz-Alonso P. M. Molfese P. J. Kuo W. J. Bick A. Frost S. J. Hancock R. Wu D. H. Mencl W. E. Duñabeitia J. A. Lee J. R. Oliver M. Zevin J. D. Hoeft F. Carreiras M. Tzeng O. J. L. Pugh K. R. Frost R. (2015). Universal brain signature of proficient reading: Evidence from four contrasting languages. Proceedings of the National Academy of Sciences of the United States of America, 112, 15510–5. 10.1073/pnas.1509321112 26621710 PMC4687557

[bibr36-03010066251364208] Rumelhart D. E. McClelland J. L. (1982). An interactive activation model of context effects in letter perception: Part 2. The contextual enhancement effect and some tests and extensions of the model. Psychological Review, 89, 60–94. 7058229

[bibr37-03010066251364208] Schoonbaert S. Grainger J. (2004). Letter position coding in printed word perception: Effects of repeated and transposed letters. Language and Cognitive Processes, 19, 333–367.

[bibr38-03010066251364208] Tan L. H. Liu H. L. Perfetti C. A. Spinks J. A. Fox P. T. Gao J. H. (2001). The neural system underlying Chinese logograph reading. NeuroImage, 13, 836–46. 10.1006/nimg.2001.0749 11304080

[bibr39-03010066251364208] Tan L. H. Spinks J. A. Eden G. F. Perfetti C. A. Siok W. T. (2005). Reading depends on writing, in Chinese. Proceedings of the National Academy of Sciences of the United States of America, 102, 8781–5. 10.1073/pnas.0503523102 15939871 PMC1150863

[bibr40-03010066251364208] Tien M. Albonico A. Barton J. J. S. (2023). Faces, English words and Chinese characters: A study of dual-task interference in mono- and bilingual speakers. Experimental Brain Research, 241, 1131–1144. 10.1007/s00221-023-06580-2 36856801 PMC9975443

[bibr41-03010066251364208] Tso R. V. Y. Au T. K. F. Hsiao J. H. W. (2014). Perceptual expertise: Can sensorimotor experience change holistic processing and left-side bias? Psychological Science, 25, 1757–67. 10.1177/0956797614541284 25085866

[bibr42-03010066251364208] Tso R. V. Y. Au T. K. F. Hsiao J. H. W. (2022). Non-monotonic developmental trend of holistic processing in visual expertise: The case of Chinese character recognition. Cognitive Research: Principles and Implications, 7, 39. 10.1186/s41235-022-00389-3 35524920 PMC9079196

[bibr43-03010066251364208] Tso R. V. Y. Chan R. T. C. Chan Y. F. Lin D. (2021). Holistic processing of Chinese characters in college students with dyslexia. Scientific Reports, 11, 1973. 10.1038/s41598-021-81553-5 33479393 PMC7820259

[bibr44-03010066251364208] Tso R. V. Y. Chan R. T. C. Hsiao J. H. W. (2020). Holistic but with reduced right-hemisphere involvement: The case of dyslexia in Chinese character recognition. Psychonomic Bulletin & Review, 27, 553–562. 10.3758/s13423-020-01721-y 32144579

[bibr45-03010066251364208] Ventura P. Delgado J. Guerreiro J. C. Cruz F. Rosário V. Farinha-Fernandes A. Domingues M. Sousa A. M. (2020a). Further evidence for a late locus of holistic word processing: Exploring vertex effect in the word composite task. Attention, Perception & Psychophysics, 82, 3259–3265. 10.3758/s13414-020-02113-z 32864728

[bibr46-03010066251364208] Ventura P. Fernandes T. Leite I. Almeida V. B. Casqueiro I. Wong A. C. N. (2017). The word composite effect depends on abstract lexical representations but not surface features like case and font. Frontiers in Psychology, 8, 1036. 10.3389/fpsyg.2017.01036 28676783 PMC5476921

[bibr47-03010066251364208] Ventura P. Fernandes T. Leite I. Pereira A. Wong A. C. N. (2019). Is holistic processing of written words modulated by phonology? Acta Psychologica, 201, 102944. 10.1016/j.actpsy.2019.102944 31704548

[bibr48-03010066251364208] Ventura P. Fernandes T. Pereira A. Guerreiro J. C. Farinha-Fernandes A. Delgado J. Ferreira M. F. Faustino B. Raposo I. Wong A. C. N. (2020b). Holistic word processing is correlated with efficiency in visual word recognition. Attention, Perception & Psychophysics, 82, 2739–2750. 10.3758/s13414-020-01988-2 32077067

[bibr49-03010066251364208] Wang J. Deng Y. Booth J. R. (2019). Automatic semantic influence on early visual word recognition in the ventral occipito-temporal cortex. Neuropsychologia, 133, 107188. 10.1016/j.neuropsychologia.2019.107188 31499046

[bibr50-03010066251364208] Wheeler D. D. (1970). Processes in word recognition. Cognitive Psychology, 1, 59–85.

[bibr51-03010066251364208] Wimmer H. Ludersdorfer P. Richlan F. Kronbichler M. (2016). Visual experience shapes orthographic representations in the visual word form area. Psychological Science, 27, 1240–8. 10.1177/0956797616657319 27435995 PMC5017316

[bibr52-03010066251364208] Wong A. C. N. Bukach C. M. Hsiao J. Greenspon E. Ahern E. Duan Y. Lui K. F. H. (2012). Holistic processing as a hallmark of perceptual expertise for nonface categories including Chinese characters. Journal of Vision, 12, 7. 10.1167/12.13.7 23220578

[bibr53-03010066251364208] Wong A. C. N. Bukach C. M. Yuen C. Yang L. Leung S. Greenspon E. (2011). Holistic processing of words modulated by reading experience. PloS One, 6, e20753. 10.1371/journal.pone.0020753 PMC3116835 21698240

[bibr54-03010066251364208] Yovel G. Tambini A. Brandman T. (2008). The asymmetry of the fusiform face area is a stable individual characteristic that underlies the left-visual-field superiority for faces. Neuropsychologia, 46, 3061–8. 10.1016/j.neuropsychologia.2008.06.017 18639566

[bibr55-03010066251364208] Zhao M. Bülthoff H. H. Bülthoff I. (2016). Beyond faces and expertise: Facelike holistic processing of nonface objects in the absence of expertise. Psychological Science, 27, 213–22. 10.1177/0956797615617779 26674129 PMC4750070

