# Supplementary material for: Reading ability underlies the composite effect for Arabic words
Source: Perception. 2025 Aug 14;54(12):948–61. doi: 10.1177/03010066251364208 (PMC12614910; doi:10.1177/03010066251364208)
Supplement: sj-pdf-1-pec-10.1177_03010066251364208 - Supplemental material for Reading ability underlies the composite effect for Arabic words [file sj-pdf-1-pec-10.1177_03010066251364208.pdf]

Supplemental material

Word lists

		Same		Different	
Set	Target	Congruent	Incongruent	Congruent	Incongruent
1	basket	basket	basics	marble	market
2	reward	reward	rewind	cowboy	coward
3	active	active	actors	nature	native
4	couple	couple	coupon	temper	temple
5	legend	legend	legion	attack	attend
6	divide	divide	divine	resume	reside
1	عريف	عريف	شريف	شريك	عريض
2	سائل	سائل	قائل	قاصر	سارح
3	جمال	جمال	مقال	مقاس	جميع
4	جواب	جواب	صواب	صوار	جواد
5	مروج	مروج	خروج	خراب	مريب
6	زميل	زميل	عميل	عميق	زمان

Left cue condition. When words were cued on the right, the words in the incongruent-same and incongruent-different words were swapped, see text for details.

Table 1. List of English and Arabic words used in the four conditions.

Prepared using sagej.cls
